# Acute administration of lithium, but not valproate, modulates cognitive judgment bias in rats

**DOI:** 10.1007/s00213-014-3847-0

**Published:** 2014-12-25

**Authors:** Rafal Rygula, Joanna Golebiowska, Jakub Kregiel, Malgorzata Holuj, Piotr Popik

**Affiliations:** 1 Affective Cognitive Neuroscience Lab, Department of Behavioral Neuroscience and Drug Development, Institute of Pharmacology, Polish Academy of Sciences, 12 Smetna Street, 31-343 Krakow, Poland; 2 Faculty of Health Sciences, Collegium Medicum, Jagiellonian University, Michałowskiego 12, 31-126 Krakow, Poland

**Keywords:** Lithium, Valproate, Cognitive judgment bias, Rat, Ambiguous cue, Animal model

## Abstract

**Rationale and objectives:**

Both valproic acid (VPA) and lithium (LI) are well-established treatments for therapy of intense and sustained mood shifts, which are characteristics of affective disorders, such as bipolar disorder (BP). As mood and cognitive judgment bias have been found to be strongly interrelated, the present study investigated, in an animal model, whether acute treatment with VPA or LI could affect cognitive judgment bias.

**Methods:**

To accomplish this goal, two groups of rats received single injections of either VPA or LI after initial behavioral training and were subsequently tested with the ambiguous-cue interpretation (ACI) test. Both drugs were administered in three doses using the fully randomized Latin square design.

**Results:**

VPA (100, 200, and 400 mg/kg) had no significant effect on the interpretation of the ambiguous cue. LI at the lowest dose (10 mg/kg) had no effect; at an intermediate dose (50 mg/kg), it significantly biased animals towards positive interpretation of the ambiguous cue, and at the highest dose (100 mg/kg), it impaired the ability of animals to complete the test.

**Conclusion:**

To our knowledge, this is the first study demonstrating lithium’s effects on increased optimistic judgment bias. Future studies may focus on the ability of putative pharmacotherapies to modify the cognitive judgment bias dimension of patients at risk for bipolar disorder or depression.

## Introduction

Although over 60 years have elapsed since its effects on mania were first described, lithium (LI) is still a mainstay in the treatment of mood disorders and remains the standard against which new mood-stabilizing or thymoleptic drugs are measured (Price and Heninger 1994). LI treatment paradoxically relieves both mania and depression—conditions that appear to be opposites, and additionally, it has been reported to have specific antisuicidal effects (Baldessarini et al. 2006; Guzzetta et al. 2007). However, the mechanism by which LI effectively treats bipolar disorder (BP) and depression and reduces the risk of suicidal behaviors is largely unknown.

Another well-established treatment for bipolar disorder is valproic acid (VPA). The drug is increasingly used for therapy of bipolar and schizoaffective disorders and neuropathic pain and for prophylactic treatment of migraines (Johannessen 2000; Johannessen and Johannessen 2003); however, similar to LI, its mode of action, especially on the systems level, remains unknown.

To date, limited research has examined effects of LI and VPA on cognitive processes in laboratory animals. Recent studies, for instance, reported that LI but not VPA significantly reduced impulsivity of mice in the three-choice serial reaction time task (Ohmura et al. 2012) and in the delay discounting task (Halcomb et al. 2013). Important insights regarding the mechanism of action of these drugs may arise from understanding the effects of LI and VPA on cognitive biases associated with affective disorders, which themselves are closely interrelated with mood. Optimistic cognitive judgment bias, for instance, is a prominent endophenotype of bipolar mania. For example, manic patients consistently display over-optimistic judgment bias leading to high-risk behaviors, such as extravagant shopping, sexual adventures, or improbable commercial schemes (Belmaker and Bersudsky 2004; Young et al. 2011). Clinical studies have highlighted the importance of excessive optimism in this disorder (Carver and Johnson 2009; Gamma et al. 2008; Giovanelli et al. 2013; Johnson 2005; Johnson and Jones 2009; Leahy 1999), and Beck and Weishaar (1995) argued that “overly optimistic expectations provide vast sources of energy and drive the manic individual into continuous goal-directed activity.” Similarly, pessimistic cognitive judgment bias is a well-known endophenotype associated with depression. According to the cognitive model proposed by Beck, negatively biased acquisition and processing of information plays a primary role in the development and maintenance of depressive disorder (Beck 1967, 1987, 2008). In this model, negative and pessimistic processing of one’s self and context, including interpretation and judgment of environmental stimuli, become pervasive (Clark et al. 1999).

Given that cognitive judgment biases are characteristics of many psychopathologies, it is surprising how little attention they have received in preclinical research. Although a number of studies over the past decade have reported that “optimism” and “pessimism” can be induced in animals following different behavioral and pharmacological manipulations (Bateson et al. 2011; Bethell et al. 2007; Brilot et al. 2010; Doyle et al. 2011; Enkel et al. 2010; Harding et al. 2004; Mendl et al. 2010; Rygula et al. 2012), very few of these studies (Enkel et al. 2010; Richter et al. 2012) have investigated cognitive judgment bias in the context of psychiatric disorders and their treatment.

The present study has been designed to investigate the effects of acute administration of two mood-stabilizing drugs that are widely used in the treatment of affective disorders—Li and VPA—on the valence of cognitive judgment bias in rats. To accomplish this goal, after initial behavioral training, different groups of rats received single injections of either LI or VPA, and they were subsequently evaluated by the ACI test. In this procedure, rats are trained to press a lever in an operant conditioning chamber to receive a food reward that is contingent on one tone and to press another lever in response to a different tone to avoid punishment by a mild electric foot-shock. The tones, which serve as discriminative stimuli, acquire positive and negative valence, and the training continues until the rats accomplish a stable, correct discrimination ratio. After attaining stable discrimination performance, the animals are tested. Ambiguous cue testing is composed of a discrimination task, as described above, consisting of the presentation of additional tones with intermediate frequencies (between positive and negative tones). The lever press response pattern to the ambiguous cue is considered an indicator of the rat’s expectation of a positive or negative event, in other words, as optimism or pessimism, respectively (for details, see (Enkel et al. 2010; Papciak et al. 2013; Rygula et al. 2012, 2013)).

Because both VPA and LI are well-established treatments for therapy of mood disorders and because mood and cognitive judgment bias have been found strongly interrelated, we hypothesized that acute LI and/or VPA treatments would change cognitive judgment bias in rats.

## Experimental procedures

### Ethics statement

These experiments were conducted in accordance with the NIH Guide for the Care and Use of Laboratory Animals and approved by the Ethics Committee for Animal Experiments at the Institute of Pharmacology Polish Academy of Sciences.

### Subjects and housing

In total, 56 (16 in the ACI experiment with VPA, 16 in the ACI experiment with LI and 24 in an additional experiment aimed at investigation of the effects of LI on shock sensitivity) male *Sprague-Dawley* rats (Charles River, Germany) weighing between 175 and 200 g (±6 weeks old) upon arrival were used in this study. The rats were group-housed (4 rats/cage) in standard type IV polycarbonate cages (59 × 38 × 20 cm) in a temperature-controlled room (21 ± 1 °C) with 40–50 % humidity under a 12/12-h light/dark cycle (lights on at 06:00 h). In all ACI experiments, the rats were mildly food restricted to approximately 85 % of their free feeding weights. This was achieved by providing 15–20 g of food per rat per day (standard laboratory chow). The food restriction started 1 week prior to training. Water was freely available, except during the test sessions. The behavioral procedures and testing were performed during the light phase of the light/dark cycle by following the protocol originally described by Enkel et al. (2010) and our previous experiments (Papciak et al. 2013; Rygula et al. 2012, 2014b).

### Apparatus

The behavioral tasks (ACI training and testing) were performed in eight computer-controlled operant conditioning chambers (MedAssociates, St Albans, Vermont, USA), and each chamber was equipped with a light, a speaker, a liquid dispenser (set to deliver 0.1 ml of 5 % sucrose solution), a grid floor through which scrambled electric shocks (0.5 mA) could be delivered, and two retractable levers. The levers were located at opposite sides of the feeder. All of the behavioral protocols, including the data acquisition and recordings, were programmed in Med State notation code (Med Associates). The experimental procedures for the ACI test used in this study were modified versions of the procedures previously described by Enkel and colleagues (2010) and have been described elsewhere (Papciak et al. 2013; Rygula et al. 2012, 2013, 2014b).

### Behavioral training

#### Positive tone training

During this phase, the rats were trained to press the lever located on the left side of the feeder to receive the sucrose solution when a tone (50 s, 2000 Hz at 75-dB sound pressure level (SPL)) signaled reward availability. Due to its association with a palatable reward, this tone acquired a positive valence and was referred to as the “positive tone,” and the associated lever was referred to as the “positive lever.” Reliable active lever pressing for the reward was achieved in three training steps: (a) presentation of the positive tone (lasting 50 s) co-occurred with a constant (every 5 s for 5 s) delivery of the sucrose solution and was followed by a 10-s intertrial interval (ITI); (b) presentation of the positive tone co-occurred with a left lever extension and was followed by a 10-s ITI (each lever press during the tone was continuously rewarded by sucrose solution delivery); and (c) was similar to (b) with the exception that after the first lever press and reward delivery, the tone was terminated and followed by a 10-s ITI. Each training session lasted for 30 min, and the training sessions continued until the animals attained a stable performance on each of the training steps (more than 200 responses maintained over three consecutive training sessions during step b and a minimum of 90 % of responses to the positive lever following positive tone presentation maintained over three consecutive sessions during step c). Positive tone training was followed by negative tone training.

#### Negative tone training

During this stage, the rats were trained to press the lever located on the right side of the feeder to avoid an electric shock (0.5 mA, 10 s) when another tone (9000 Hz at 75 dB SPL) signaled a forthcoming punishment. Due to its association with a concomitant punishment, this tone acquired a negative valence and was referred to as the “negative tone.” The associated lever was referred to as the “negative lever.” A reliable active lever press avoidance response was achieved in two training steps: (a) the presentation of the negative tone was accompanied by the occurrence of electric shocks unless the rat pressed the right (negative) lever, which terminated the shock and tone presentation, and (b) the presentation of the negative tone preceded the occurrence of the electric shocks. The delay from the tone onset to the electric shock occurrence was progressively increased from 1 to 40 s. Pressing the negative lever before the shock onset terminated the tone and began a 10-s ITI (avoidance response). Pressing the negative lever after the shock onset terminated the tone and shock and was referred to as the “escape response.” The maximum duration of the tone/shock-application was 50 s (i.e., 40 s of tone presentation followed by 10 s of a tone/shock co-occurrence), and the tone presentations were separated by 10-s ITIs. Daily training sessions consisted of 40 tone presentations and lasted 30 min. The animals had to accomplish at least 60 % correct avoidance responses maintained over three consecutive training sessions before proceeding to the discrimination training.

#### Discrimination training

During this phase, the rats were trained to discriminate between positive and negative tones by responding to the appropriate levers (as learned in previous training stages) to maximize reward and minimize punishment delivery. The tones, which consisted of 20 positive and 20 negative tones, were presented in a non-systematic order and separated by 10-s ITIs. Pressing the positive lever during the positive tone presentation resulted in an instant reward delivery and initiated the ITI. Pressing the negative lever during the negative tone presentation resulted in a negative tone termination and initiated the ITI. Pressing the wrong lever (e.g., pressing the left lever instead of the right lever in response to a negative tone presentation), as well as escape responses or response omissions, were considered failed trials. Each training session lasted 40 min. Animals had to minimally achieve 70 % correct responses with each lever maintained over 3 consecutive discrimination sessions to proceed to the ACI test—a learning criterion adapted from the seminal paper by Enkel and colleagues (2010).

### Ambiguous-cue testing

The ACI testing session consisted of 20 positive, 20 negative, and 10 intermediate (ambiguous) tone presentations. The frequency of the intermediate tones was set to 5000 Hz at 75 dB. This frequency was selected on the basis of the protocol described by Enkel and colleagues (2010) and was confirmed to be intermediate in terms of the response pattern in a pilot experiment (data not shown). Each test lasted 50 min. The tones were presented in a pseudo-randomized order and separated by 10-s ITIs. Any lever press during the ambiguous tone presentation terminated the tone but had no consequences. If the rat did not respond within 50 s of the ambiguous tone presentation, the tone was terminated, and a response omission was scored.

During ACI testing, the responses to each tone (positive, ambiguous, and negative) were scored and analyzed as the proportion of the overall number of responses to a given tone. The proportion of omissions was analyzed separately. To calculate the optimism index, the proportion of negative responses to the ambiguous cues was subtracted from the proportion of positive responses, resulting in values ranging between −1 and 1, where values above 0 indicate an overall positive judgment and an optimistic interpretation of the ambiguous cue.

### Drug treatment

Both drugs were purchased from Sigma-Aldrich (Poznan, Poland). Valproic acid sodium salt (100, 200, and 400 mg/kg) and lithium chloride (10, 50, and 100 mg/kg) were dissolved in physiological saline and administered intraperitoneally (i.p.) in a dose volume of 1 ml/kg 30 min before the ACI and shock sensitivity test sessions. The effects of each drug treatment were tested in separate ACI experiments using 16 (VPA) and 16 (LI) animals. Initially, the animals within each cage were randomly assigned to different treatment groups. The drugs were tested using fully randomized Latin square designs. The wash out period between administrations of different drug doses in the Latin square design was 1 week. Each group of animals was treated with only one drug. All of the drug doses used in this study were similar to those previously shown to be effective in rodent models of BP mania (Frey et al. 2006a, b, c).

### Evaluation of the effects of LI on shock sensitivity

An additional experiment aimed at investigation of the effects of LI on shock sensitivity was performed on a group of 24 experimentally naïve rats. In this experiment, on day 1 (baseline), the animals were exposed to 20 scrambled foot shocks (0.2 mA, 10 s) delivered with a variable ITI. During this stage, the animals learned that shocks could be avoided by escaping to the opposite compartment of the experimental apparatus and their baseline performance was recorded and used to match further experimental groups. On day 2 (test), the animals that received injections of either physiological saline or one of the three doses of LI were tested in the same experimental design but this time, the shock intensity was identical to this used in the ACI experiments (0.5 mA, 10 s). The experiment was performed in four computer-controlled shuttle boxes (MedAssociates, St Albans, Vermont, USA) where each box (44 × 21 × 18 cm) was divided in two equal-sized compartments. During baseline and test sessions, the rats were allowed to move freely from one compartment to another at any time. The position of the animal was tracked by eight photocells in each of the boxes. If a rat moved from one compartment to another upon receiving the foot shock (analogous to the one used in the ACI experiments), this response was recorded as an escape. If the rat did not escape during the 10 s of shock delivery, the trial was terminated and was recorded as a failure. The procedure was repeated 20 times during the baseline and test sessions with a variable ITI of 20–40 s. The main measure of the shock sensitivity was the number of escape responses made during the experimental session.

### Statistics

The data were analyzed using SPSS (version 20.0, SPSS Inc., Chicago, IL, USA). The effects of drugs on the optimism index were investigated using repeated measures analysis of variance (ANOVA) with the within-subjects factor of dose (four levels: control, dose 1, dose 2, and dose 3). The effects of drugs on the processing of ambiguous cues and reference tones were investigated using three-way repeated measures ANOVA with within-subject factors of dose (four levels: control, dose 1, dose 2, and dose 3), lever (two levels: positive and negative), and tone (three levels: positive, ambiguous, and negative). The effects of LI on shock sensitivity were investigated using two-way repeated-measures ANOVA with within-subject factor of treatment (two levels: baseline and test) and between-subject factor of dose (four levels: control, dose 1, dose 2, and dose 3). For pair-wise comparisons, the values were adjusted by using Sidak’s correction factor for multiple comparisons (Howell 1997). All of the tests of significance were performed at *α* = 0.05. For repeated-measures analyses, the sphericity was verified by using Mauchly’s test. The data are presented as the means ± SEM.

## Results

All trained rats (16 for the experiment with LI and 16 for the experiment with VPA) reached the criterion of at least 70 % correct responses with each lever, maintained over three consecutive discrimination sessions, and qualified for cognitive bias testing. The animals that were used in the experiment with LI reached the criteria of positive tone, negative tone, and discrimination trainings after 5.6 ± 0.38, 7.9 ± 1.3, and 28.8 ± 1.0 days, respectively, whereas the group used in the experiment with VPA reached the criteria after 5.2 ± 0.3, 20.1 ± 1.7, and 30.1 ± 2.1 days, respectively.

### Valproic acid

**Fig. 1:**
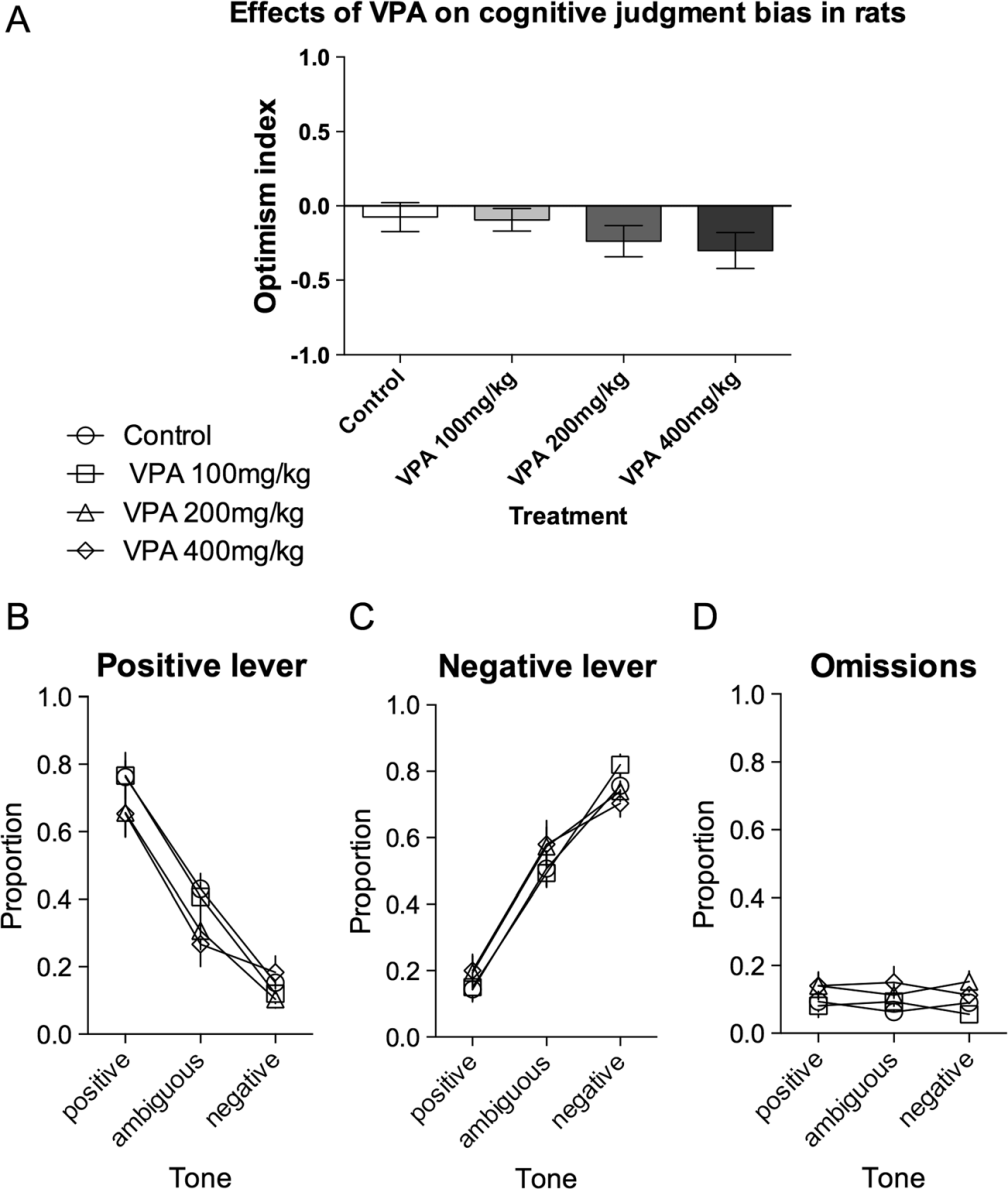
Effects of three different doses of VPA on the judgment bias of rats in the ambiguous-cue interpretation test. **a** The mean ± SEM optimism index of the control (*white bar*) and VPA 100 mg/kg (*light grey bar*), 200 mg/kg (*grey bar*), and 400 mg/kg (*black bar*) treated rats. The mean ± SEM proportion of **b** positive, **c** negative, and **d** omitted responses to the trained and ambiguous tones in the control (*white circle*) and VPA 100 mg/kg (*white square*), 200 mg/kg (*white triangle*), and 400 mg/kg (*white diamond*) treated rats. *N* = 16/group

As shown in Fig. 1a, VPA (100, 200 and 400 mg/kg) did not have significant effects on the optimism index of rats (*F*_(3,45)_ = 1.79, NS). Although further analysis of the lever responses revealed a significant dose × lever × tone interaction (*F*_(6,90)_ = 2.22, *p* < 0.05), post hoc analysis revealed a significant (*p* < 0.05) difference only between the effects of the doses of 100 and 400 mg/kg on responding to the negative lever after negative tone presentation (Fig. 1c). Analysis of the proportion of the trials in which the animals did not respond to the ambiguous and unambiguous tones (after different doses of VPA) revealed no significant differences between experimental groups (Fig. 1d, no significant effect of dose (*F*_(3,45)_ = 2.15, NS) or dose × tone interaction (*F*_(6,90)_ = 1.26, NS)).

### Lithium chloride

**Fig. 2:**
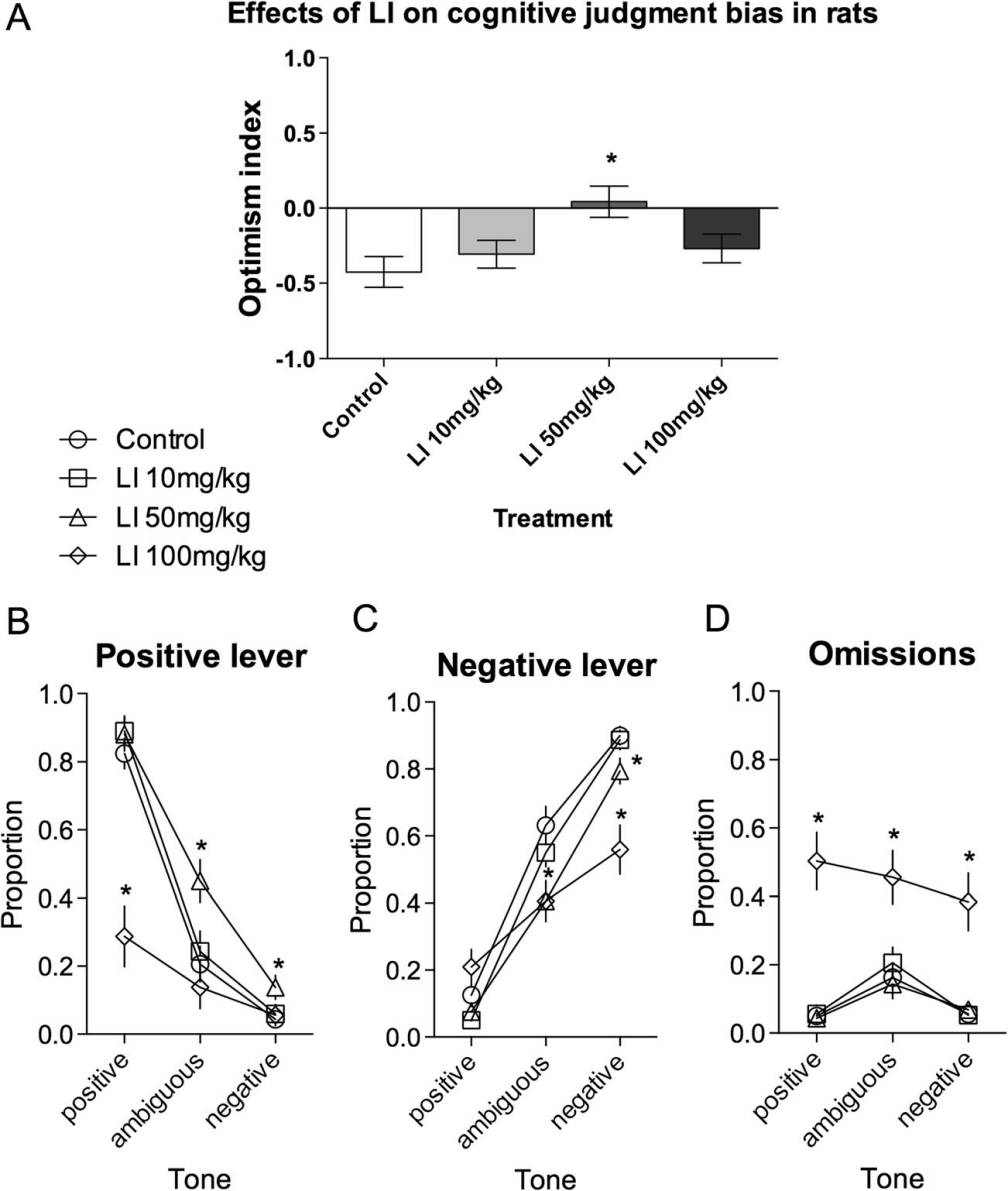
Effects of three different doses of LI on the judgment bias of rats in the ambiguous-cue interpretation test. **a** The mean ± SEM optimism index of the control (*white bar*) and LI 10 mg/kg (*light grey bar*), 50 mg/kg (*grey bar*), and 100 mg/kg (*black bar*) treated rats. The mean ± SEM proportion of **b** positive, **c** negative, and **d** omitted responses to the trained and ambiguous tones in the control (*white circle*) and LI 10 mg/kg (*white square*), 50 mg/kg (*white triangle*), and 100 mg/kg (*white diamond*) treated rats. *Asterisk* indicates significant (*p* < 0.05) differences between the control and LI treated animals. *N* = 16/group

As shown in Fig. 2a, LI at the lowest (10 mg/kg) and highest (100 mg/kg) tested doses exerted no significant effects on the cognitive judgment bias index of rats. However, administration of the middle dose (50 mg/kg) made rats significantly (*p* < 0.01) more optimistic (repeated-measures ANOVA (*F*_(3,45)_ = 4.87, *p* < 0.05)).

As shown in Fig. 2b–c, this positive/optimistic response bias observed in rats after LI administration resulted from the significantly (*p* < 0.05) increased number of positive lever responses in response to ambiguous and negative tones and the significantly (*p* < 0.05) decreased number of negative lever responses following the presentation of ambiguous and negative tones (significant dose × lever × tone interaction (*F*_(6,90)_ = 20.36, *p* < 0.001)).

**Fig. 3:**
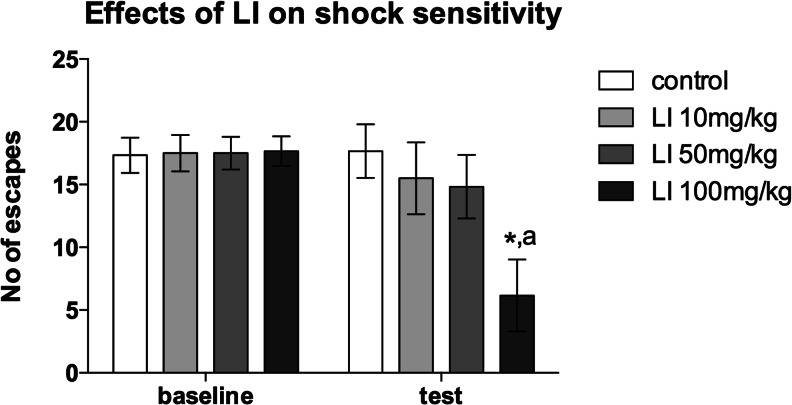
Effects of three different doses of LI on the shock sensitivity in the simple shock avoidance paradigm. The mean ± SEM number of escape responses made by the control (*white bar*) and LI 10 mg/kg (*light grey bar*), 50 mg/kg (*grey bar*), and 100 mg/kg (*black bar*) treated rats. *Asterisk* indicates significant (*p* < 0.05) differences between the control and LI treated animals. “*a*” indicates significant (*p* < 0.001) differences between the baseline and test in LI 100 mg/kg treated animals. *N* = 6/group

Analysis of the proportion of trials in which the animals did not respond revealed only that after the highest dose of LI, the rats made significantly (*P* < 0.05) more response omissions in response to all (reference and ambiguous) tones rather than after saline treatment (significant dose × tone interaction (*F*_(6,90)_ = 3.09, *p* < 0.01, Fig. 3)).

### Effects of LI on shock sensitivity

As shown in Fig. 3, LI at the lowest (10 mg/kg) and intermediate (50 mg/kg) tested doses exerted no significant effects on the number of shock escapes made by animals in the simple shock avoidance paradigm. However, administration of the highest dose (100 mg/kg) significantly decreased the number of shock escapes as compared to the baseline (*p* < 0.05) and to controls (*p* < 0.001). Repeated-measures two-way ANOVA revealed significant treatment × dose interaction (*F*_(3,20)_ = 3.93, *p* < 0.05).

## Discussion

In the present study, using the recently developed ambiguous-cue interpretation test, we investigated whether acute treatment with LI or VPA could affect cognitive judgment bias in an animal model. We showed that acute LI administration (at the dose of 50 mg/kg) significantly increases optimistic judgment bias in rats, whereas administration of another mood stabilizer, VPA, does not have such an effect. To our knowledge, this is the first study to assess the effects of mood stabilizing drugs on cognitive judgment bias in rodents.

It is noteworthy that, at the lowest dose, LI administration had no significant effects on the responses to either reference (positive and negative) tones or ambiguous cues. At the intermediate dose, LI selectively affected lever pressing in response to the ambiguous cue only by increasing the proportion of positive lever presses and decreasing the proportion of negative lever presses, while at the highest dose, a general decrease in responding to all types of tones was observed. This pattern clearly shows that, similar to clinical conditions, the effects of LI administration on cognitive judgment bias in rodents are limited to a certain dose range, with low doses being ineffective and the highest doses causing toxic effects. Notably, as shown in the simple avoidance paradigm, the highest dose of LI rendered animals less sensitive to the electric foot shocks, which might have influenced their performance also in the ACI test.

Although arguably, LI is one of the most valuable drugs in the treatment of affective disorders, an understanding of its mechanisms of action has lagged behind its clinical utility. Recent studies in humans (Swann et al. 2002) and in rats (Halcomb et al. 2013) have suggested that LI might exert its antimanic and antisuicidal effects by reducing impulsivity. To date, however, very limited number studies have examined the effects of LI on other cognitive processes in laboratory animals. The results of our study show that LI effectivity in the treatment of affective disorders could also be mediated by its effects on cognitive judgment bias, which, as shown recently, can be changed not only by positive or negative emotions but also (and more importantly) by acute pharmacological stimulation of the serotonergic (5-HT), noradrenergic (NA), and dopaminergic (DA) systems (Rygula et al. 2014a). Although our study did not examine the molecular targets of LI that may be responsible for its optimism-enhancing effects, previous studies have reported a variety of actions of this drug on neurotransmitter systems and second messenger cascades.

LI increases the activity of the serotonergic (5-hydroxytryptamine, 5-HT) system, modulates dopamine (DA) signaling, has well-documented effects on ϒ-aminobutyric acid (GABA) and acetylcholine (ACh) function, inhibits apoptosis, reduces oxidative stress, and promotes the transcription of neuroprotective proteins in the brain (Malhi et al. 2009, 2012). It will be of interest for future studies to assess the effects of pharmacological blockade of the known direct targets of LI on cognitive judgment bias.

We have shown recently that the valence of cognitive judgment bias in rats, similar to humans, has both enduring traits and transient state components (Rygula et al. 2013). Indeed, as also found in the present study, differences in the basal (trait) levels of cognitive judgment bias between animals could be observed; the rats used in the experiment with LI were generally pessimistic (AVG optimism index after saline administration −0.43 ± 0.1), contrary to the group used for the experiment with VPA, which was more neutral (AVG optimism index after saline administration −0.08 ± 0.1). Since only the animals used in the LI experiment showed basal pessimistic judgment bias, it is unlikely that it might have resulted from the stress associated with repeated injections or mild electric shocks delivered during ACI testing because in that case, the effect would be also present in the VPA experiment.

We suggest the possibility that the modality/direction of the mood-stabilizing effects of LI may be dependent on the valence of cognitive judgment bias, e.g., by increasing optimistic judgment bias in ‘pessimists’ and increasing pessimistic judgment bias in optimists. Clearly, further studies investigating how individual differences in the interpretation of the ambiguous cue interact with LI effects are required to test this hypothesis.

There are at least two limitations of the present study that have to be mentioned. The first is that we only assessed the effects of acute administration of LI or VPA. We cannot exclude that prolonged administration of both drugs could potentiate their effects and reveal other aspects of their action on cognitive judgment bias. However, considering the widely reported toxicity of prolonged LI administration (Malhi et al. 2009, 2012), chronic treatment with this drug would require monitoring of the drug blood levels, which currently could not be performed in our laboratory. Moreover, although indeed, the clinical efficacy of LI is mainly observed following the prolonged treatment, to our knowledge, there are no clinical/experimental data regarding the time course of its effects on cognitive judgment bias neither in humans nor animals. Thus, our study is the first to evaluate the effects of LI on this domain. The rapid onset of LI action reported here constitutes important result per se, indicating that the effects of this drug on cognitive processes may not require chronic administration.

The second limitation is that for the assessment of the effects of LI and VPA on cognitive judgment bias, we used naïve animals. It would be more insightful to test the effects of archetypal treatment for bipolar mania in an animal model of this condition. However, as we have shown recently, chronic administration of psychostimulant drugs such as amphetamine or cocaine, considered the gold standard for modeling mania in rodents, does not change the cognitive judgment bias of rats (Rygula et al. 2014b). The mentioned study would require invention of different, perhaps genetic, models of dopaminergic hyperactivity.

As discussed elsewhere (Rygula et al. 2012), contrary to previous studies (Enkel et al. 2010; Harding et al. 2004), we did not investigate different degrees of ambiguity. The experimental design with only one ambiguous tone allowed us to apply a reasonably high (10) number of stimuli in only one testing session, and the 50 discriminations per session provided a broad and precise scale for the response valence.

Taken together, using the recently developed ACI test, we demonstrated for the first time that acute LI administration induces an optimistic shift in the cognitive judgment bias in rats. We also showed that this effect is specific to a narrow dose range. This same effect was not observed with acute valproate treatment. Our findings clearly show that LI may exert its therapeutic action by influencing cognitive judgment bias.

## References

[CR1] Baldessarini RJ, Pompili M, Tondo L (2006). Suicide in bipolar disorder: risks and management. CNS Spectr.

[CR2] Bateson M, Desire S, Gartside SE, Wright GA (2011). Agitated honeybees exhibit pessimistic cognitive biases. Curr Biol.

[CR3] Beck AT (1967). Depression: clinical, experimental, and theoretical aspects.

[CR4] Beck AT (1987). Cognitive models of depression. J Cogn Psychother.

[CR5] Beck AT (2008). The evolution of the cognitive model of depression and its neurobiological correlates. Am J Psychiatry.

[CR6] Beck AT, Weishaar ME, Corsini RJ, Wedding D (1995). Cognitive therapy. Current psychotherapies.

[CR7] Belmaker RH, Bersudsky Y (2004). Bipolar disorder: mania and depression. Discov Med.

[CR8] Bethell EJ, Holmes A, Maclarnon A, Semple S (2007). Evidence that emotion mediates social attention in rhesus macaques. PLoS One.

[CR9] Brilot BO, Asher L, Bateson M (2010). Stereotyping starlings are more ‘pessimistic’. Anim Cogn.

[CR10] Carver CS, Johnson SL (2009). Tendencies toward mania and tendencies toward depression have distinct motivational, affective, and cognitive correlates. Cogn Ther Res.

[CR11] Clark DA, Beck AT, Alford BA (1999). Scientific foundations of cognitive theory and therapy of depression.

[CR12] Doyle RE, Lee C, Deiss V, Fisher AD, Hinch GN, Boissy A (2011). Measuring judgement bias and emotional reactivity in sheep following long-term exposure to unpredictable and aversive events. Physiol Behav.

[CR13] Enkel T, Gholizadeh D, von Bohlen Und Halbach O, Sanchis-Segura C, Hurlemann R, Spanagel R, Gass P, Vollmayr B (2010). Ambiguous-cue interpretation is biased under stress- and depression-like states in rats. Neuropsychopharmacology.

[CR14] Frey BN, Andreazza AC, Cereser KM, Martins MR, Valvassori SS, Reus GZ, Quevedo J, Kapczinski F (2006). Effects of mood stabilizers on hippocampus BDNF levels in an animal model of mania. Life Sci.

[CR15] Frey BN, Andreazza AC, Rosa AR, Martins MR, Valvassori SS, Reus GZ, Hatch JP, Quevedo J, Kapczinski F (2006). Lithium increases nerve growth factor levels in the rat hippocampus in an animal model of mania. Behav Pharmacol.

[CR16] Frey BN, Valvassori SS, Reus GZ, Martins MR, Petronilho FC, Bardini K, Dal-Pizzol F, Kapczinski F, Quevedo J (2006). Effects of lithium and valproate on amphetamine-induced oxidative stress generation in an animal model of mania. J Psychiatry Neurosci.

[CR17] Gamma A, Angst J, Ajdacic-Gross V, Rossler W (2008). Are hypomanics the happier normals?. J Affect Disord.

[CR18] Giovanelli A, Hoerger M, Johnson SL, Gruber J (2013). Impulsive responses to positive mood and reward are related to mania risk. Cogn Emot.

[CR19] Guzzetta F, Tondo L, Centorrino F, Baldessarini RJ (2007). Lithium treatment reduces suicide risk in recurrent major depressive disorder. J Clin Psychiatry.

[CR20] Halcomb ME, Gould TD, Grahame NJ (2013). Lithium, but not valproate, reduces impulsive choice in the delay-discounting task in mice. Neuropsychopharmacology.

[CR21] Harding EJ, Paul ES, Mendl M (2004). Animal behaviour: cognitive bias and affective state. Nature.

[CR22] Howell DC (1997). Statistical methods for psychology.

[CR23] Johannessen CU (2000). Mechanisms of action of valproate: a commentatory. Neurochem Int.

[CR24] Johannessen CU, Johannessen SI (2003). Valproate: past, present, and future. CNS Drug Rev.

[CR25] Johnson SL (2005). Mania and dysregulation in goal pursuit: a review. Clin Psychol Rev.

[CR26] Johnson SL, Jones S (2009). Cognitive correlates of mania risk: are responses to success, positive moods, and manic symptoms distinct or overlapping?. J Clin Psychol.

[CR27] Leahy RL (1999). Decision making and mania. J Cogn Psychother.

[CR28] Malhi GS, Adams D, Berk M (2009). Is lithium in a class of its own? A brief profile of its clinical use. Aust N Z J Psychiatr.

[CR29] Malhi GS, Tanious M, Das P, Berk M (2012). The science and practice of lithium therapy. Aust N Z J Psychiatr.

[CR30] Mendl M, Brooks J, Basse C, Burman O, Paul E, Blackwell E, Casey R (2010). Dogs showing separation-related behaviour exhibit a ‘pessimistic’ cognitive bias. Curr Biol.

[CR31] Ohmura Y, Tsutsui-Kimura I, Kumamoto H, Minami M, Izumi T, Yamaguchi T, Yoshida T, Yoshioka M (2012). Lithium, but not valproic acid or carbamazepine, suppresses impulsive-like action in rats. Psychopharmacol (Berl).

[CR32] Papciak J, Popik P, Fuchs E, Rygula R (2013). Chronic psychosocial stress makes rats more ‘pessimistic’ in the ambiguous-cue interpretation paradigm. Behav Brain Res.

[CR33] Price LH, Heninger GR (1994). Lithium in the treatment of mood disorders. N Engl J Med.

[CR34] Richter SH, Schick A, Hoyer C, Lankisch K, Gass P, Vollmayr B (2012). A glass full of optimism: enrichment effects on cognitive bias in a rat model of depression. Cogn Affect Behav Neurosci.

[CR35] Rygula R, Pluta H, Popik P (2012). Laughing rats are optimistic. PLoS One.

[CR36] Rygula R, Papciak J, Popik P (2013). Trait pessimism predicts vulnerability to stress-induced anhedonia in rats. Neuropsychopharmacology.

[CR37] Rygula R, Papciak J, Popik P (2014a) The effects of acute pharmacological stimulation of the 5-HT, NA and DA systems on the cognitive judgement bias of rats in the ambiguous-cue interpretation paradigm. Eur Neuropsychopharmacol J Eur Coll Neuropsychopharmacol 10.1016/j.euroneuro.2014.01.012 24503278

[CR38] Rygula R, Szczech E, Kregiel J, Golebiowska J, Kubik J, Popik P (2014b) Cognitive judgment bias in the psychostimulant-induced model of mania in rats. Psychopharmacol (Berl) 10.1007/s00213-014-3707-y PMC4302237 25116482

[CR39] Swann AC, Bowden CL, Calabrese JR, Dilsaver SC, Morris DD (2002). Pattern of response to divalproex, lithium, or placebo in four naturalistic subtypes of mania. Neuropsychopharmacology.

[CR40] Young JW, van Enkhuizen J, Winstanley CA, Geyer MA (2011). Increased risk-taking behavior in dopamine transporter knockdown mice: further support for a mouse model of mania. J Psychopharmacol.

